#  Biopharmacology of Enzyme Conjugates: Vasoprotective Activity of Supramolecular Superoxide Dismutase-Chondroitin Sulfate-Catalase Derivative 

**Published:** 2010

**Authors:** A.V. Maksimenko, A.V. Vavaev, L.I. Bouryachkovskaya, V.P. Mokh, I.A. Uchitel, V.L. Lakomkin, V.I. Kapelko, E.G. Tischenko

**Affiliations:** Institute of Experimental Cardiology, Russian Cardiology Research-and-Production Complex

**Keywords:** antioxidant therapy, superoxide dismutase, catalase, chondroitin sulfate, vascular wall, oxidative stress, hydrogen peroxide, bienzyme conjugate, platelets, ring arterial fragment, hemodynamics, vasoprotective activity

## Abstract

Bienzyme conjugate was obtained by the covalent connection of superoxide dismutase with catalase through endothelial glycocalyx glycosaminoglycan – chondroitin sulfate (SOD-CHS-CAT). This SOD-CHS-CAT conjugate has vasoprotective activity in respect to platelet interactions, tonus of the ring arterial fragment of a rat blood vessel, as well as normalization of hemodynamic parameters in rats and rabbits in conditions of oxidative stress caused by the administration of hydrogen peroxide. The SOD-CHS-CAT conjugate had antiplatelet potential due to its antiaggregation action manifested through the combination of enzyme activities and an acquired supramolecular structure. The influence on arterial fragment tonus was equivalent for SOD and CAT in native and conjugated form. Blood pressure and heart rate were significant and effectively normalized with SOD-CHS-CAT conjugate in rats and rabbits (after hydrogen peroxide administration as a perturbance stimulus). We have discovered the possibility of using the antioxidant bienzyme conjugate in chronic prophylaxis. It is important for a real development of the oral form of the SOD-CHS-CAT conjugate. These results indicate that the development of enzyme conjugates can be medically significant, as a promising approach for the creation of new drugs.

##  Introduction 

Enzymes are widely used as medication in thrombolytic therapy [1, 2]. The “golden molecule” of fibrinolysis and “golden time” of thrombolysis are known to increase the arsenal of biocatalysts used in treatment [3, 4]. However, the therapeutic applications of enzymes are far from having been exhausted, and there is a search for new forms that can be used in the development of original treatments for various pathologies [5, 6].

It is widely known today that the development of many pathologies is accompanied by oxidative stress [7, 8]. Normally, reactive oxygen species (ROS) participate in cellular signaling. However, the distortion of the balance between oxidative and antioxidant activity, as well as the mass production of ROS, results in oxidative stress. Excess ROS in the organism leads to the modification of macromolecules, disbalance of metabolic pathways and progression of the pathologic processes [9] that can be prevented or delayed by means of antioxidant administration [10]. Enzymes are considered as very efficient antioxidants, since they are specific and their mode of action is known in most cases [11]. Oxidative stress is known to play an important role in the pathogenesis of most cardiovascular disorders, which is why many cardiology studies are focused on antioxidants.

 Current views hold that superoxide dismutase, catalase, and glutathione peroxidase are the main antioxidant enzymes of the body. The autonomous functioning of the first two makes them attractive in terms of the development of antioxidant medication to protect the cardiovascular system against oxidative stress. Based on the described advantages, we chose Cu,Zn-super­oxide dismutase (SOD), and catalase for the development of an enzymatic antioxidant derivate. 

Our approach was based on biochemical coupling of SOD and CAT activity, when the product of SOD-reaction (H _2_ O _2_ ) is used as a substrate by CAT, which finally yields water and oxygen as products [9, 11]. Accumulation of a glycosaminoglycan of endothelial glycocalyx – chondroitin sulfate (CHS) – in zones of initial atherosclerotic changes of vessels (i. e. in sites of potential damage of the vascular wall) [12] allowed the use of CHS as a cross-linking modifier of the enzyme subunits [13]. The water-soluble form of the obtained exogenic enzymatic conjugate SOD-CHS-CAT can be administered intravenously and per orally. It should be noted that the dimensions of a CAT molecule are 10.5 × 10.5 × 5.0 nm [14], and they are 6.7 × 3.6 × 3.3 nm for a SOD molecule [15]. The polymer chain of CHS winds around the enzymatic subunits and connects them, thus forming a covalent conjugate, which was confirmed by means of electrophoresis under denaturing conditions [13, 16]. Moreover, the conjugate is more active *in vivo * than combinations of individual components, which are accounted for by its optimal intravascular distribution and higher action efficiency [17]. Based on its dimensions ─ (17–20) × (14–18) × (8–12) – the conjugate occupies the lower zone of the nano-scale and, therefore, can be considered a nanoparticle. It is thought that the physical, chemical, and biological properties of molecular objects with nano-dimensions would surprisingly differ from the properties of their individual components, in part due to their quantum-mechanical effects.

 Considering the supramolecular SOD-CHS-CAT conjugate, a bienzymatic nano-device, in the current work we studied its interactions with platelets (occurring in the blood stream) and with a ring arterial fragment (occurring on the surface of the vascular wall). We also studied the properties of the supramolecular conjugate at the level of the organism in laboratory animals, both under conditions of oxidative stress as modeled by infusion of hydrogen peroxide, and under stress-free conditions. 

##  Experimental procedures 

**Materials**

In the present study, we used the following reagents: Cu,Zn-superoxide dismutase (SOD), isolated from bovine red blood cells (specific activity 3,000 U/mg protein); catalase (CAT) from bovine liver (specific activity 11,000 U/mg protein); chondroitin-4-sulfate A (molecular weight 25-50 kDa) from bovine trachea; benzoquinone, dimethylformamide, β-galactosidase (from *Escherichia coli* ), xantine, hydrogen peroxide, noradrenaline (NA), N _ω_ -nitro- *L* -arginine (L-NNA), acetylcholine, sodium nitroprusside (SNP) were purchased from Sigma, USA. Xantinoxidase was purchased from Calbiochem (USA), nitrotetrazolium blue - from Reanal (Hungary), sephadex G-25 and sephacryle S-300 - from Pharmacia (Sweden). The other reagents were of analytical grade purity and manufactured in Russia.

Bienzyme SOD-CHS-CAT conjugate was obtained as previously described [16]. The protein content in a SOD-CHS-CAT preparation was 4-6% weight, specific SOD activity was 60 U/mg solid, and CAT - 140 U/mg solid. For preparation of the SOD-CHS-CAT conjugate with irreversibly inactivated enzymes, SOD and CAT were preliminarily incubated in the presence of 0.3 M hydrogen peroxide (pH 7.0, 0.02 М phosphate buffer, 3 hours at room temperature) and at pH 11.8-12.0 (0.05 M NaOH, 2 hours at room temperature), respectively [16].

**Methods**

*Biochemical measurements.* The protein content in preparations was determined by the Bradford method. The enzymatic activity of SOD was measured as inhibition of reduction of nitrotetrasolium blue in the system xantine-xantinoxidase, pH 7.8 [13]. CAT activity was determined spectrophotometrically as a decrease in absorption at λ=240 nm (disappearance of hydrogen peroxide; pH 7.0, room temperature) [16].

*Studies on platelet aggregation* . In order to investigate the effects of hydrogen peroxide and SOD-CHS-CAT on platelet aggregation, we used the blood of volunteers, which was taken from the ulnar vein and stored in plastic tubes containing 0.13 M sodium citrate (pH 7.3). Platelet-rich plasma (PRP) was obtained by centrifugation of blood samples at 180g for 15 min. Platelet aggregation was estimated by means of the laser two-channel aggregation analyzer BIOLA (LA 230-2 model, NPF Biola, Russia). Apart from the customary approach based on registration of light transmission (Born’s method), platelet aggregation was measured based on the analysis of fluctuations of the light transmission. The relative value of these fluctuations is proportional to the average radius of aggregates and allows to study the formation of micro-aggregates; i. e., those containing less than 100 platelets. It also allows the omission of the possible uncertainties that arise from light absorption by plasma and changes in platelet shape, which is of primary importance in studies of spontaneous aggregation.

 The ability to form small-sized aggregates (3-100 platelets) was estimated by measuring both spontaneous aggregation and aggregation induced by the addition of 0.5 μM ADP, 0.5 μM serotonine and 1.0 μM TRAP (thrombin receptor-activation peptide) by the method of registration of the avarage size of the aggregates (in relative units). The formation of large aggregates (more than 100 platelets) in response to the addition of 5.0 μM ADP and 6.0 μM TRAP was estimated by the Born’s method in % light transmission. Measurements were carried out during 2 hours after blood samples were obtained in 0.3 ml cuvettes. 

Platelet adhesion was estimated by means of electronic microscopy. A 15 μM sample was mixed with a saline solution, H _2_ O _2_ and/or CAT preparations and loaded onto an adhesive surface (glass), incubated 15 minutes at room temperature in an enclosed space for preventing drying-out, rinsed with saline to wash the unattached platelets off and fixed by 2.5% glutaraldehyde for 1.5 hours. After fixation, the samples were dehydrated and prepared for electron microscopy.

 Platelets of various shapes were quantified on 25 scanning fields at a magnification of 2500x (PHILLIPS PSEM 550x scanning electron microscope). Adhesion was expressed as a percentage of platelets stuck to the surface. 

*Investigation of changes in tone of rat ring arterial fragment. * After the decapitation of Wistar rats (males, 350-400 g), the abdominal cavity was opened and abdominal aorta was isolated. The isolated aortal segment was cleared from connective tissue and sliced into ring fragments 3 mm in length. Aortal fragments were then put on stainless needles connected to the tensosensor (which measures the mN forces produced by the arterial fragment) and placed into a Krebs-Henseleit buffer insufflated with carbogenum at 37 ^o^ C and pH 7.4 [18]. Oxidative stress was modeled by the addition of hydrogen peroxide to the preparation previously subjected to NA preconstriction (preliminary constriction of the arterial fragment induced by the addition of NA prior to another stimulus). Alteration of vascular tone was estimated relative to the level of contraction induced by 0.1 μM NA taken as 100%. Various concentrations of antioxidant enzyme derivatives were added 10 minutes before the addition of hydrogen peroxide against a background of NA-preconstriction. The production of endogenous NO and its influence on the tone of the ring arterial fragment was initiated by the addition of the exogenous inhibitor of NO-syntase L-NNA (0.1 μM).

*Experiments in vivo. * Tolerance and the protective action of the SOD-CHS-CAT conjugate under conditions of H _2_ O _2_ -induced oxidative stress were studied in male rabbits (n=29) 3.65±0.10 kg in weight and male Wistar rats (n=13) 427±7g in weight. All animals were anaesthetized prior to the experiments by infusion of ketamine.

 In rabbits undergoing ketamine narcosis (50-60 mg/kg weight), catheters (PE-50 diameter) were placed in the central artery of one ear and the marginal veins of both ears. After the initial narcosis, (55 mg/kg) 5% ketamine was infused with a syringe pump (SAGE Instruments, USA) at a rate of 36-54 μl/hour per 1 kg of weight. The vein of the other ear was bolus-infused with saline or SOD-CHS-CAT solution, and 0.8% hydrogen peroxide solution at a rate of 0.4 ml/min for 3 min, performed twice with a 20 min interval, which was necessary for full recovery of parameters after the first injection of hydrogen peroxide and distribution of the conjugate in the body. In the acute experiment, the infusion of reagents was done in the following order: hydrogen peroxide - saline solution (control group) or SOD-CHS-CAT (experiment group) - hydrogen peroxide. Registration of the mean blood pressure (BP), heart rate (HR) and electrocardiogram (ECG) in the second lead was carried out on BIOGRAF-4 (Saint-Petersburg State University of Aerospace Instrument Making, Russia) equipped with an ADC board NI 6210 (National Instruments, USA). Physiological signals were processed using corresponding software (Dr. E.V. Lukosh­kova). After the registration of initial BP, HR and ECG (during 15 min) animals were infused with hydrogen peroxide (3 min) and the aforementioned physiological parameters were measured again during 10 minutes. The total dose of hydrogen peroxide injected throughout the experiment was 0.31 μM/kg. The number of animals in the control group was 12; in the experimental group - 15. 

 In Wistar rats under ketamine narcosis (100 mg/kg), catheters (PE-50) were placed in the carotid artery and jugular vein. The protocol of the experiment was essentially similar to the abovementioned, but with a minor difference: the time of hydrogen peroxide infusion was extended by 2 minutes in order to obtain similar effects on hemodynamic. The total dose of hydrogen peroxide injected in rats was 4.5 μM/kg. No animals died in the course of these experiments. 

 A separate series of experiments on rabbits (n=8) was focused on the investigation of tolerance of various doses of the SOD-CHS-CAT conjugate (therapeutic – 1.5 mg/kg and also 7.5 and 15 mg/kg). HR, BP, and ECG parameters were measured in these experiments. After a 15-minute control registration of BP, HR (taken as 100%), and ECG, the first dose of SOD-CHS-CAT (1.5 mg/kg ) was injected and HR, BP, and ECG were registered during 15 min. Afterward, the next dose of SOD-CHS-CAT (7.5 mg/kg) was injected, followed by the registration of hemodynamic parameters. 20 minutes later, another (15 mg/kg) dose, ten times greater than the therapeutic dose, was administered and hemodynamic parameters were measured. The animals that received a total dose of SOD-CHS-CAT 16 times greater in 72 hours were used to estimate the preventive effects of the bienzyme conjugate during oxidative stress (as described above). This contrasts with the effects obtained in both acute and control experiments on animals that had not been administered anything beforehand. 

*Statistical treatment of obtained results* . The results obtained are presented as a mean value±standard error, where *n* is the number of animals. The two groups were compared using the two-sample Student’s t-test (the statistical significance of the differences was estimated at *p * < 0.01). In the case of more than two comparison groups, the statistical calculations were carried out using the ANOVA ( *p * < 0.01) method.

**Fig. 1:**
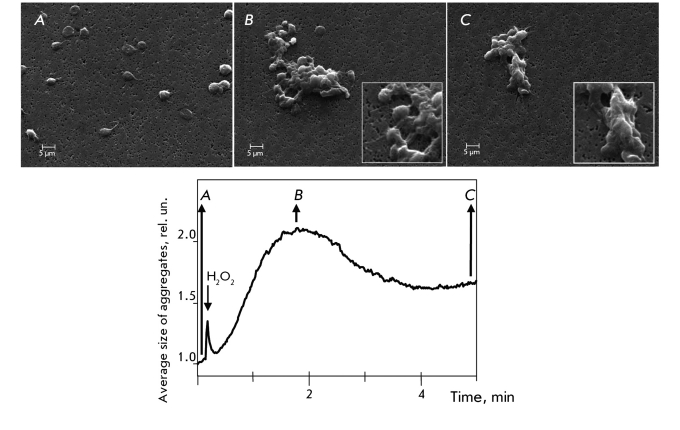
Typical platelet aggregation curve induced by 50 μM – 2.0 mM hydrogen peroxide and composition of platelet aggregates in different stages of their formation. A: segregate platelets before aggregation, mainly, in discoid forms; B: sample of aggregation peak – aggregates consist of tight core in their center and weakly attached platelets on their periphery (friable structure of peripheral area has been shown on insert in magnified view); C: sample after five minutes of platelet aggregation – the size of aggregates is decreased, weakly attached platelets are absent on periphery, strong fusion of platelets in aggregates. Magnification x2500, on insert x5000.

##  Results and Discussion 

**The effects of SOD-CHS-CAT on platelets**

**Fig. 2:**
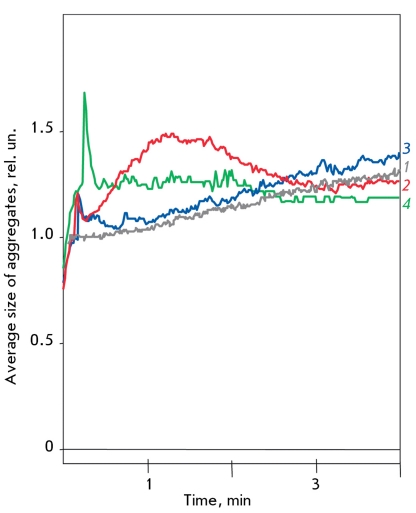
Influence of hydrogen peroxide, CAT and SOD-CHS-CAT on platelet aggregation. Curve of spontaneous platelet aggregation (1), aggregation in presence of 300 μM hydrogen peroxide (2), addition of 3,000 U native CAT first, followed by 300 μM hydrogen peroxide (3), addition of 400 U CAT activity of SOD-CHS-CAT first, followed by 300 μM hydrogen peroxide (4).

Under normal conditions, hydrogen peroxide acts as an intra- and inter-cellular signaling molecule. In the concentration range of 20-50 μM, hydrogen peroxide has limited cytotoxicity for many cell types. Under physiological conditions, 50 μM concentration is considered high [19]. The addition of 50-2000 μM hydrogen peroxide *in vitro * led to an aggregation of platelets (Fig. 1). Scanning electronic microscopy showed that at the moment of maximum aggregation the aggregates consisted of tightly packed platelets in the core. However, the platelets were bound rather loosely on the periphery (Fig. 1b). By the 5 ^th^ minute in the process, the average size of the aggregates had decreased and their structure had become so dense that it was impossible to distinguish discrete platelets (Fig. 1C). The decrease in the size of the aggregates could be explained by a dissociation of weakly bound platelets from aggregate clusters, as well as the consolidation of aggregate cores. The addition of 3,000 U native catalase to the cuvette of the aggregometer substantially lowered the aggregation induced by 300 μM hydrogen peroxide (Fig. 2). The SOD-CHS-CAT conjugate was shown to suppress aggregation in a dose as low as 400 U CAT activity, showing an increased dose-dependent antioxidant activity compared to CAT alone. Neither CAT nor CHS affects platelet aggregation.

**Fig. 3:**
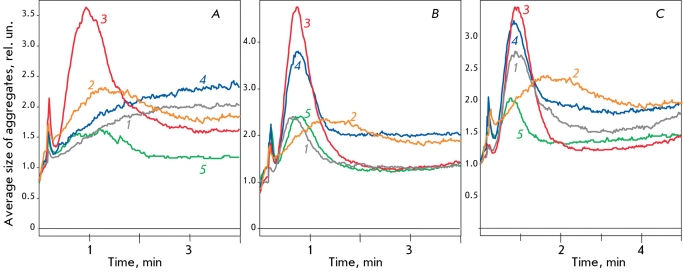
Influence of CAT and SOD-CHS-CAT on platelet aggregation induced by ADP (A), serotonin (B), and TRAP (C) in the presence of hydrogen peroxide. A: Platelet aggregation curves in the presence of 0.5 μM ADP (1), 300 μM hydrogen peroxide (2), 0.5 μM ADP, and 300 μM hydrogen peroxide (3); preventive administration of 3,000 U CAT and then 0.5 μM ADP with 300 μM hydrogen peroxide (4); and preventive administration of 400 U of CAT activity of SOD-CHS-CAT and then 0.5 μM ADP with 300 μM hydrogen peroxide (5). B: Platelet aggregation curves in the presence of 0.5 μM serotonin (1), 300 μM hydrogen peroxide (2), 0.5 μM serotonin, and 300 μM hydrogen peroxide (3); preventive administration of 3,000 U CAT and then 0.5 μM serotonin with 300 μM hydrogen peroxide (4); and preventive administration of 400 U of CAT activity of SOD-CHS-CAT and then 0.5 μM serotonin with 300 μM hydrogen peroxide (5). C: Platelet aggregation curves in the presence of 1 μM TRAP (1), 300 μM hydrogen peroxide (2), 1 μM TRAP, and 300 μM hydrogen peroxide (3); preventive administration of 3,000 U CAT and then 1 μM TRAP with 300 μM hydrogen peroxide (4); and preventive administration of 400 U of CAT activity of SOD-CHS-CAT and then 0.5 μM TRAP with 300 μM hydrogen peroxide (5). Typical aggregation curves were representative of 4-5 experiments.

The presence of 300 μM H _2_ O _2 _ increased platelet activation, in terms of their aggregation, induced by various inducers (with varying mechanisms of action, i.e., ADP, serotonin and TRAP (Fig. 3)). The addition of 3,000 U CAT or 400 U CAT-activity of the SOD-CHS-CAT conjugate to PRP suppressed the activating effects of H _2_ O _2_ (curves 4 and 5 in Figs. 3A, 3B and 3C, respectively). Application of the conjugate proves its increased antioxidant activity, exceeding that of CAT. The effect demonstrated dose-dependence and reached a maximum (which did not substantially change when increased doses were added to the cuvette) at 3,000 and 400 U CAT activity for CAT and SOD-CHS-CAT, respectively.

**Fig. 4:**
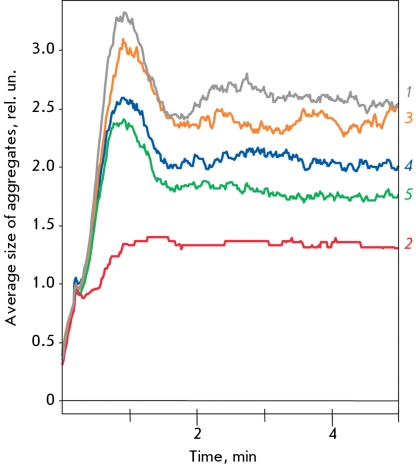
Influence of SOD-CHS-CAT on platelet aggregation induced by 0.5 μM ADP. Aggregation curves with 0.5 μM ADP (1), and preventive administration (in equimolar concentration) of corresponding 400 U CAT activity of SOD-CHS-CAT forms: SOD-CHS-CAT (2), SOD_inact_-CHS-CAT_inact_ (3), SOD-CHS-CAT_inact _(4), SOD_inact_-CHS-CAT (5). Typical aggregation curves were representative of 3-5 experiments.

Due to the catalase activity, the CAT and SOD-CHS-CAT derivates suppressed the effects of H _2_ O _2_ on platelets (Figs. 2 and 3). However, it was shown that the platelets themselves can produce ROS [20]. This is in line with the inhibiting effect of the SOD-CHS-CAT on ADP-induced platelet aggregation (Fig. 4). At the same time, the effect of the CAT-CHS derivate was quite moderate, and CAT did not possess it at all (data not shown). The anti-aggregate activity of the bienzyme conjugate was due to the presence of enzymatic activities (curves 1 and 2 in Fig. 4) and also to its unique supramolecular structure, mediated by CHS (curves 1 and 3 in Fig. 4) [11, 17]. Indeed, β-galactosidase (similar in molecular size to SOD-CHS-CAT) used in the same experimental scheme in equimolecular protein concentrations did not inhibit the ADP-induced platelet aggregation.

**Fig. 5:**
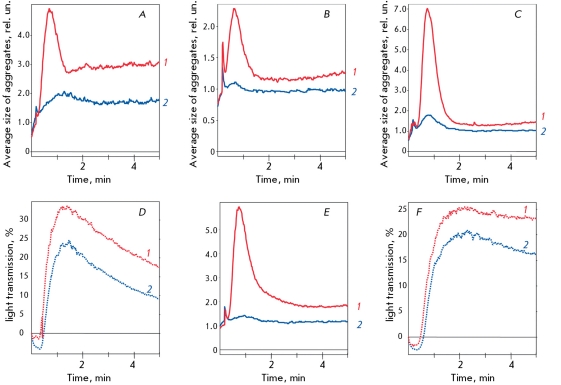
Influence of SOD-CHS-CAT conjugate (400 U of CAT activity, curve 2) on platelet aggregation induced (curve 1) by: 0.5 μM (A) and 5.0 μM (D) ADP; 0.5 μM (B) and 5.0 μM (E) serotonin; 1 μM (C) and 6 μM (F) TRAP. Typical aggregation curves were representative of 3-6 experiments. Abscissa is time (minutes), ordinate is average size of platelet aggregates (in rel. units, A-C, E) or light transmission (%, D and F).

The action of SOD-CHS-CAT was expressed in inhibiting ADP-, serotonin-, and TRAP-induced platelet aggregation (all the inducers exploit different mechanisms of action and were used in different concentrations) (Fig. 5). This is a new quality for the SOD-CHS-CAT conjugate, since the individual components lack it.

**Fig. 6:**
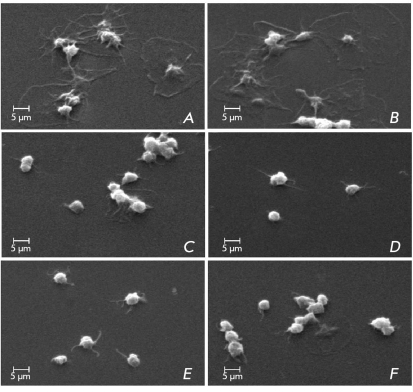
Electron microscopy picture of platelet adhesion on glass surface (Scanning Electron Microscope Phillips PSEM 550x, magnification x2500). Platelet adhesion on glass with addition to PRP: saline (physiological solution, A), hydrogen peroxide (B), SOD-CHS-CAT (D). Adhesion of platelets on glass preventively treated by SOD-CHS-CAT and then the addition on the glass surface of the PRP with hydrogen peroxide (C); by hydrogen peroxide and then the addition on the glass surface of PRP with SOD-CHS-CAT (E) or with native CAT (F).

Platelet spreading on the adhesive surface is one of the critical stages of hemostasis, which induces a sequence of reactions, leading to thrombus formation. Platelet adhesion and spreading takes place during transferral to glass (Fig. 6a). In the presence of H _2_ O _2, _ the amount of spread platelets increases (Fig. 6b), but when SOD-CHS-CAT conjugate is transferred beforehand to the glass (Fig. 6c) or the PRP (Fig. 6d), no spread platelets are found in the sample. A similar situation can be observed when PRP and SOD-CHS-CAT are transferred to glass which had H _2_ O _2_ added to it beforehand (Fig. 6e). When PRP and CAT are added, the amount of spread platelets decreases significantly (Fig. 6f). We note that free CHS did not demonstrate anti-aggregate inhibition with regard to hydrogen peroxide. It is probable that this effect of the bienzyme conjugate has something to do with its adhesive and antioxidant qualities, which allow it, on the one hand, to protect the surface from platelet adhesion, and, on the other, to neutralize H _2_ O _2_ , which strengthens adhesion and platelet spreading on glass.

 The results we obtained indicate that the bienzyme SOD-CHS-CAT conjugate expresses an antioxidant dose-dependent effect during induced platelet aggregation in the presence of hydrogen peroxide. The anti-aggregate activity of the SOD-CHS-CAT conjugate is reliably higher than that of other CAT derivates and is demonstrated in a wide range of conditions when platelet aggregation is stimulated by various inducers (ADP, serotonin, TRAP – all of which differ in terms of mechanisms of action), both with and without hydrogen peroxide. The latter is evidence of a new aspect of the anti-aggregate potential of SOD-CHS-CAT, absent in the native enzymes and free CHS, and determined by its molecular composition and size. These qualities of the bienzyme SOD-CHS-CAT nanoconjugate show that it has a promising future in biopharmaceutical development for purposes of antioxidant therapy. They are also proof of the promises in using enzyme conjugates as medicinal agent. 

**The change in the tone of a ring fragment of rat aorta**

The effects of oxidative stress on the tone of a ring fragment of rat abdominal aorta were modeled by the addition of H _2_ O _2 _ against a background of NA preconstriction. The latter made up 50-60% of the maximum possible contraction of the arterial fragment, which made it possible to measure both contraction and relaxation. The change in vascular tone was estimated relative to the contraction exhibited in response to 0.1 µM NA, taken at a 100% range of interval (for the gradation of the size of tone change), and at the baseline of the experiment as 0% (to indicate the direction of tone change, contraction or relaxation). Hydrogen peroxide caused a dose-dependent vascular contraction. At a 0.01 mM concentration of H _2_ O _2_ , we observed a small (10-12%) tone increase. At a 0.1 mM H _2_ O _2_ , this increase was more (48-50%) with a subsequent decrease to the initial level of NA preconstriction (0-3% relaxation). At a 1.0 mM concentration of H _2_ O _2_ , there was a rapid contraction of the arterial fragment (88-90%), which was followed by a relaxation phase (68-70%). Thus, the model we presented revealed a dose-dependent effect of hydrogen peroxide and was suitable for an experimental study of the effect of ROS on the tone of the vascular fragment.

**Fig. 7:**
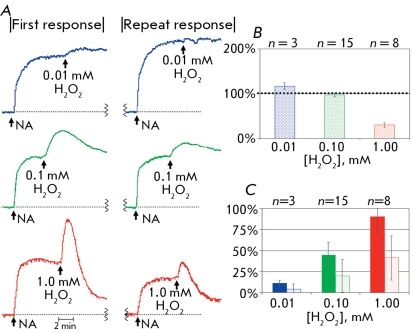
First and repeat responses to NA and hydrogen peroxide. A: the original tracings of the first and repeat responses. B: the size of repeat response to NA, the size of first contraction induced by NA is equal to 100 %. The size of first (painted bar graphs) and repeat (empty bar graphs) contraction (C) of arterial fragment after administration of different concentrations of hydrogen peroxide.

After triple washing and a 15-minute rest period, the vascular fragment was exposed to NA and hydrogen peroxide again at the concentrations used earlier (0.01-0.1 mM). The level of functional activity of the vascular fragment during the second response decreased with the increase in hydrogen peroxide concentration, both in relation to the second addition of NA, as well as to the amount of contraction during the second addition of H _2_ O _2_ (Fig. 7). The reliable decrease in the second contraction of the arterial fragment after the second addition of large concentrations of hydrogen peroxide serves as a measure of the invariability of vascular reactions.

**Fig. 8:**
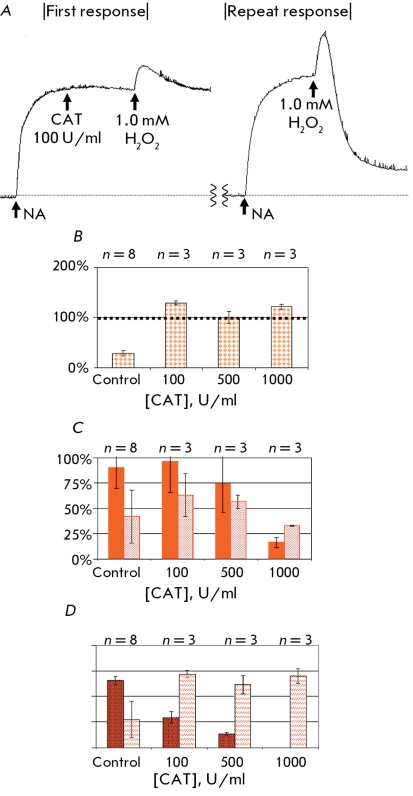
Dose-dependent influence of native CAT on tone of arterial fragment after repeat addition of NA and 1.0 mM hydrogen peroxide. A: the original tracings. B: the size of repeat response to NA after addition of different concentrations of CAT (painted bar-graphs), CAT activity is equal in twain of comparison. The size of first (painted bar-graphs) and second (empty bar-grafs) contraction (C) and relaxation (D) of ring arterial fragment to 0.1 mM hydrogen peroxide according to CAT activity, (U/ml).

The addition of native CAT against the background of NA-induced preconstriction did not influence the vascular tone in any of the concentrations used. The reaction to the addition of 1.0 mM H _2_ O _2 _ in the presence of CAT decreased substantially in terms of contraction and relaxation. At a concentration of 1,000 U/ml the CAT effect of 1.0 mM H _2_ O _2_ decreased to 16% contraction. CAT displayed a dose-dependent protective effect against the stimulation of the vascular fragment by hydrogen peroxide.

Prior incubation of the arterial fragment with CAT substantially influenced the invariability of subsequent responses to NA and hydrogen peroxide (Fig. 8). At a CAT concentration of 100 U/ml and higher the size of the second response to NA did not change, whereas in the absence of the enzyme it decreased three- or fourfold (Fig. 8b). The presence of CAT during the first addition of H _2_ O _2_ increased the subsequent contractive and dilative response (in comparison with the control), and when 1,000 U/ml CAT was used the second response reliably exceeded the first (Figs. 8c and 8d). Insofar as vascular reactions to the repeated effects of NA and H _2_ O _2_ in the absence of CAT decrease significantly, the protective function of CAT is obvious. The data received after using 0.1 mM H _2_ O _2 _ for 25-500 U/ml CAT confirm this conclusion.

**Fig. 9:**
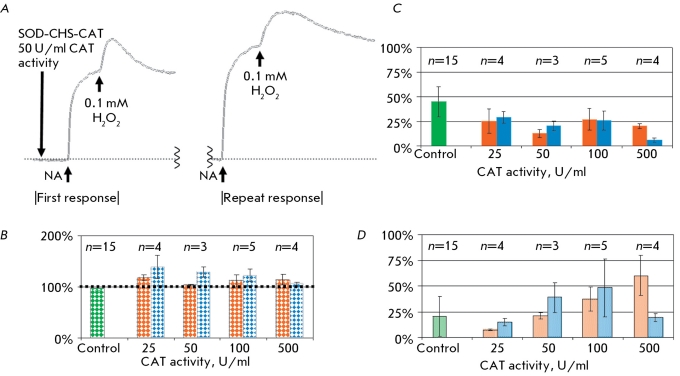
Comparative effects of protective action of CAT and SOD-CHS-CAT against 0.1 mM hydrogen peroxide. A: the original tracings. B: the size of repeat response to NA after the addition of different concentrations of CAT (dark bar-graphs) and SOD-CHS-CAT (gray bar-graphs), CAT activity is equal in twain of comparison. C and D: the contraction of rat aorta fragment for first (C) and repeat (D) responses to 0.1 mM hydrogen peroxide in the presence (in first response) of noted concentration of CAT (left in twain) and SOD-CHS-CAT (right in twain) according to CAT activity (U/ml).

The preventive character of antioxidants implies their defensive effect as soon as the physiological level of ROS is exceeded. Therefore, we used 0.1 mM H _2_ O _2 _ to estimate the comparative effectiveness of the protective effect of CAT and the SOD-CHS-CAT conjugate in our experimental model when testing the invariability of vascular functionality (Fig. 9). CAT derivates were used in concentrations that were identical in terms of CAT activity (U/ml). Native CAT and the SOD-CHS-CAT conjugate maintained their level of response to the secondary addition of NA (Fig. 9b); this addition is comparable to the control indicators. In a concentration range of 25-100 U/ml of catalase activity, the CAT and SOD-CHS-CAT derivates demonstrated similar (in terms of size) protective effects against 0.1 mM H _2_ O _2_ . At a concentration of 500 U/ml, the SOD-CHS-CAT conjugate lowered the amplitude response to H _2_ O _2 _ more effectively than CAT. The protective effect expressed by SOD-CHS-CAT may be connected to its affinity to the vascular wall (due to the conjugation of SOD with CAT via glycosaminoglycan of endothelial glycocalyx [11, 17]) and/or to the presence of SOD and endogenous NO in the model system. In the system described above, it is difficult to estimate the sorption of enzyme derivates because of the flow of vascular reactions not only from within outwards, but also from without inwards (i.e. on both the intima and the adventitia) [21]. The defensive effect of SOD with regard to NO, however, is possible to estimate.

**Fig. 10:**
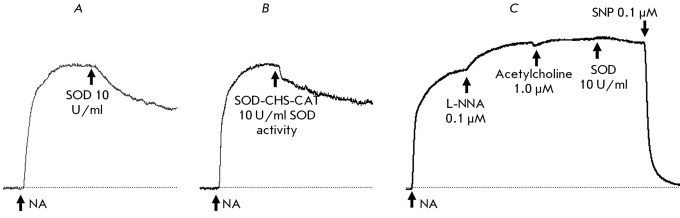
Influence of SOD activity on the tone of arterial fragment. The original tracings after administration of 10 U/ml native SOD (A) or SOD-CHS-CAT (B) with NA preconstruction. C: the alteration of vascular tone after administration of NA, N-nitro-L-arginine (L-NNA), acetylcholine, native SOD and sodium nitroprusside (SNP), respectively. Alike curves were obtained after 3-4 experiments.

This effect of native SOD and SOD-CHS-CAT was quite pronounced when studied on an NA-preconstricted arterial fragment (Fig. 10). Both substances caused reliable vascular relaxation. This suggests that the observed dilation was determined by the conservation of endogenous NO. This is because SOD neutralized the superoxide radical capable of turning NO into peroxynitrite, which does not have vasodilative properties. The experimental evidence of this is the inhibiting effect of NO-synthase with L-NNA, which caused an increase in vascular tone (Fig. 10c). This effect expresses the inhibition of NO-synthase activity and the development of a contractive response because of the lack of a dilative effect in endogenous NO. The introduction of acetylcholine (as an inducer of vascular relaxation by means of its effect on the endothelial receptors which start off NO-synthase) gave no response, which indicates the effective inhibition of NO-synthase. Against the background of L-NNA, the introduction of 10 U/ml SOD-activity gave no response. This confirms the role of endogenous NO in the vascular relaxation we observed earlier. The introduction of sodium nitroprusside (SNP), which acts as an NO donor, caused dilation (Fig. 10c); i.e., the vascular fragment did not lose its ability to relax under the influence of NO. The increase in the bioavailability of NO when the SOD-CHS-CAT conjugate was used, similar in size to the effect of native SOD (Fig. 10a), demonstrates the effectiveness of the vasoprotective action of the conjugate, due to the activity of its SOD component.

The bienzyme SOD-CHS-CAT overall reliably demonstrates its vasoprotective qualities that are connected to the activity of both of its enzyme components (SOD and CAT). These are no less effective than the native forms of biocatalysts. This is convincing evidence of the SOD-CHS-CAT conjugate’s potential for medicinal development. However, its safety and effectiveness need to be assessed and proven *in vivo* .

**Vasoactivity of the SOD-CHS-CAT conjugate**

*in vivo*

Intravenous bolus injection of varying doses of the SOD-CHS-CAT conjugate in rabbits showed that BP and HR were changed by no more than 4% relative to their average means in intact narcotized animals. ECG did not reveal any alterations of the ST-interval, rhythm or conductivity disturbances and other abnormalities, even when doses of the conjugate 10 times greater were administered. Singular intra-abdominal injections of SOD-CHS-CAT into BALB/c mice and CBAxC57B16 F1-hybrid mice showed a low acute toxicity of the conjugate and the absence of mutagenic properties, as confirmed through the Ames test. These data, together with the high tolerance of the SOD-CHS-CAT conjugate in animals as was mentioned earlier, as well as its pronounced antithrombotic activity [11, 17], justifies the significance of its further investigation.

**Fig. 11:**
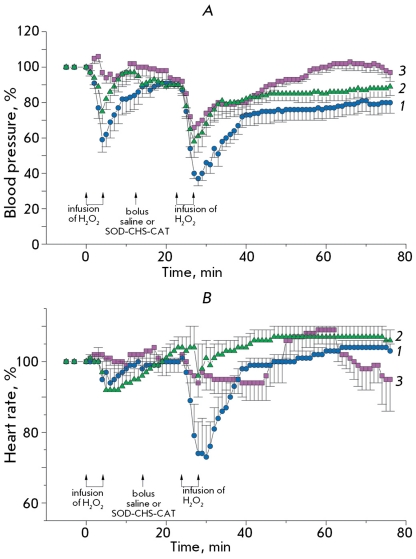
Alteration of BP (A) and HR (B, both in % from initial) of anesthetized rabbits for oxidative stress (infusion of 0.8 % hydrogen peroxide) in control (n=12, bolus administration of saline, curve 1) and test (n=9, bolus administration of SOD-CHS-CAT conjugate, curve 2) groups. The curve 3 is appertained to prophylaxis test group (n=6) of rabbits obtaining the SOD-CHS-CAT conjugate earlier (three days earlier than this experiment) with hydrogen peroxide and after its first administration. The arrows show the moment beginning and ending the infusion of hydrogen peroxide and bolus administration of saline or SOD-CHS-CAT conjugate (1.5 mg/kg).

The first injection of hydrogen peroxide into rabbits (Fig. 11A, curve 1) caused a sharp decrease in BP (up to 60% of its initial level), which was restored during 10 minutes in the control group to 90% of its initial level. Similar changes were observed in the experimental group (Fig. 11A, curve 2). It is noteworthy that the administration of the SOD-CHS-CAT derivate 72 hours prior to the experiments reliably prevented a drop in BP. In that case, we observed only minor initial oscillations in the BP level followed by smoothening, and the stabilization of BP at its initial level (Fig. 11A, curve 3). Preventive administration of SOD-CHS-CAT accounted for its prophylactic effect in respect to HR in animals (Fig. 11B, curve 3) in comparison with data obtained in the control (Fig. 11B, curve 1) and the experimental (Fig. 11B, curve 2) groups.

Ten minutes after the injection of the conjugate (time required for its distribution in the rabbit’s organism), which did not affect the characteristics of central hemodynamics, hydrogen peroxide was injected for the second time in a similar concentration and at the same rate. The second infusion caused more profound changes in BP and HR (Fig. 11). The course of BP recovery had two stages – a fast one that took 5 minutes and a slow one.

After the administration of SOD-CHS-CAT in the animals of the experimental group, the changes in BP and HR in response to the injection of the second dose of hydrogen peroxide were significantly less pronounced and recovery was faster (Fig. 11).

 After the first injection of hydrogen peroxide, we did not detect any substantial changes in ECG in rabbits, although in the control group the ST-segment was lowered. The second injection of hydrogen peroxide caused the ST-interval to rise, especially in the cases when rabbits experienced breathing problems. At 5-8 minutes, we observed both isolated and group extrasistoles. The first injection of hydrogen peroxide was often accompanied by dyspnea, while after the second injection we observed hindered breathing and bronchospasms, which were more pronounced in the control group. 

**Fig. 12:**
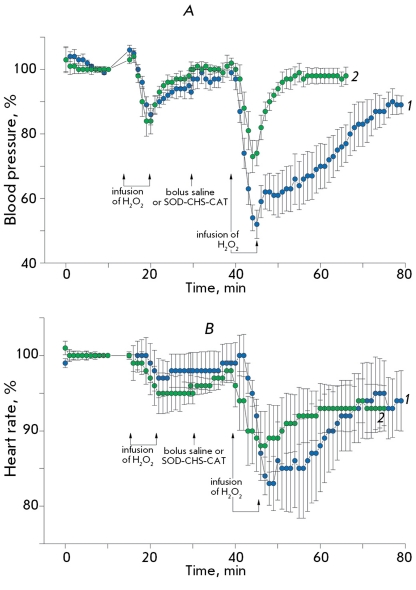
Alteration of BP (A) and HR (B, both in % from initial) of anesthetized rats. The arrows show the moment of the beginning and end of the intravenous infusion of 0.8 % hydrogen peroxide (oxidative stress) and bolus administration of saline (n=6, control group, curve 1) or SOD-CHS-CAT conjugate (n=7, test group, curve 2).

The first injection of hydrogen peroxide in rats caused an initial short-term (no more than 1 minute) 3-5% increase in BP with a subsequent decrease by 15% and full recovery during 10 minutes (Fig. 12A). Under these conditions, HR decreased by 3-5% and recovered to up to 96-98% of its initial level during 10 minutes (Fig. 12B, curves 1 and 2). Injection of SOD-CHS-CAT did not affect hemodynamics and ECG in rats. In ten minute’s time after the injection of the conjugate (time required for its distribution in the rat’s organism), which did not affect the characteristics of central hemodynamics, hydrogen peroxide was injected for the second time. The second infusion caused more profound changes in BP and HR as compared to the first one. The decrease in BP reached 52% of its initial level in the control group and 73% in the experimental group (Fig. 12A, curves 1 and 2, respectively, p < 0.05). BP recovery in the control group was significantly slower. A statistically significant difference in BP recovery in both groups was observed in the first minutes.

 The shape of the ECG signal was not significantly changed, although the number of ventricular extrasistols increased in both groups of animals, especially after the second injection of hydrogen peroxide. 

Thus, the bienzyme SOD-CHS-CAT conjugate was shown to significantly prevent changes in the hemodynamics caused by hydrogen peroxide in two animal species. The conjugate effectively neglected hydrogen peroxide’s direct influence on smooth muscle and cardiac cells [22], presumably due to the neutralisation of both superoxide and H _2_ O _2_ . The effects of SOD-CHS-CAT were reliably detected before the second injection of hydrogen peroxide, when the antioxidant systems were weakened by the first injection of H _2_ O _2_ .

The preventive effect of the SOD-CHS-CAT conjugate was more pronounced in animals that were administered high doses of the conjugate 3 days before the experiment. In this case, we observed a full recovery of BP and HR after the first injection of H _2_ O _2_ . This is the most pronounced antioxidant activity in comparison with the other cases considered. It is possible that preventive administration of the conjugates accounts for a more efficient resistance to the action of hydrogen peroxide through direct and/or indirect pathways. The importance of CAT activity is confirmed by its necessary presence as a component of lecitinized SOD-based therapy of bleomycin-induced pulmonary fibrosis in mice [23]. The dose-dependence of the therapeutic effect of lecitinized SOD is described as a bell-shaped curve. In the range of high SOD concentrations, the introduction of CAT restores the effect of SOD due to the prevention of peroxide accumulation. The presence of both SOD and CAT in the enzyme conjugate accounts for its antithrombotic activity [9, 17] and normalization of BP and HR, which were distorted by hydrogen peroxide infusion (Figs. 11 and 12). The effectiveness of oral and inhalation administration of a modified form of SOD, described in [24] and [23], indicates the importance of developing oral forms of SOD-CHS-CAT as means of preventive antioxidant therapy [25].

##  Conclusions 

 The results of the present study indicate that supra-molecular enzymatic conjugates are potentially effective and safe and that they can be used in practical medicine. Covalent cross-linking of enzymatic subunits accounts for a mutual stabilization of biocatalyst activity. Augmented molecular weight and the inclusion of chondroitin sulphate (glycosoaminoglycan of the endothelial glycocalyx) in the conjugate results in a vasoprotective activity of the SOD-CHS-CAT derivate on the inner surface of the vascular wall. The therapeutical effect of the SOD-CHS-CAT is determined by covalent coupling of SOD and CAT activities, which underlies their combined action and formation of harmless products of enzymatic conversion. The supra-molecular structure of the nanoconjugate accounts for its previously unknown quality – the capacity to prevent induced platelet aggregation. The activity of the components of the SOD-CHS-CAT conjugate is very pronounced, since the influence of the SOD and CAT activities of the conjugate on the tone of the vascular fragment is similar to that of native enzymes. The contribution of chondroitin sulphate to the antiaggregational effect is also very clear. The described qualities determine the higher antioxidant activity of the SOD-CHS-CAT conjugate, as compared to the other combinations of its individual components. The SOD-CHS-CAT conjugate is well-tolerated, it possesses satisfactorily acute toxicity, normalizing action in relation to hemodynamics under oxidative stress, and a pronounced therapeutical effect. All these factors make the conjugate a prospective drug candidate. This study represents a new approach to the development of therapeutically significant enzymatic conjugates. 
